# Six-Month Surgical Outcomes of Ahmed ClearPath™ ST250 Implantation in Seven Eyes With Refractory Glaucoma: A Case Series

**DOI:** 10.7759/cureus.115837

**Published:** 2026-09-05

**Authors:** Shiho Kasahara, Ryo Tomita, Kenya Yuki

**Affiliations:** 1 Department of Ophthalmology, Nagoya University Graduate School of Medicine, Nagoya, JPN

**Keywords:** ahmed clearpathtm st, anti-glaucoma medication, elevated intraocular pressure, glaucoma drainage device, glaucoma surgery, refractory glaucoma, small tube, tube shunt surgery

## Abstract

Purpose: The purpose of this study is to evaluate the short-term clinical outcomes of Ahmed ClearPath™ small tube (ST) 250 implantation in patients with refractory glaucoma.

Methods: This retrospective study included seven consecutive patients who underwent Ahmed ClearPath™ ST250 implantation at Nagoya University Hospital between December 2025 and April 2026. The primary outcome measure was intraocular pressure (IOP) at six months.

Results: The mean age at surgery was 68.7 ± 17.7 years, and the median age was 74 years (range: 42-87 years). Four eyes had primary open-angle glaucoma, and one eye each had glaucoma secondary to retinal detachment surgery, uveitis, and Descemet stripping automated endothelial keratoplasty. Preoperative average IOP was 35.0 ± 6.6 mmHg, and median IOP was 32.0 mmHg (range: 28.0-45.0 mmHg). After Ahmed ClearPath^TM^ ST surgery, IOP decreased to 15.7 ± 9.9 mmHg in one day, median IOP: 12.0 mmHg (range: 5.0-30.0 mmHg), 17.1 ± 12.1 mmHg, median IOP: 11.0 mmHg (range, 6.0-40.0 mmHg) in one week, 13.2 ± 8.2 mmHg, median IOP: 14.0 mmHg (range, 3.0-26.0 mmHg) in two weeks, 10.8 ± 4.1 mmHg, median IOP: 10.5 mmHg (range, 6.0-18.0 mmHg) in one month, 16.2 ± 8.0 mmHg, median IOP: 16.5 mmHg (range: 6.0-28.0 mmHg) in three months, and 11.0 ± 2.7 mmHg, median IOP: 12.0 mmHg (range: 7.0-14.0 mmHg) in six months. Stent removal was performed in five eyes on postoperative days 7, 13, 14, 90, and 90, whereas the stent remained in two eyes throughout the follow-up period. Five of seven patients were glaucoma medication-free after surgery at six months. No shallow or flat anterior chamber, choroidal detachment, suprachoroidal hemorrhage, hypotony maculopathy, or conjunctival leakage were observed. Numerical hypotony, defined as an IOP of <6 mmHg, was observed in two cases but resolved spontaneously without any specific treatment.

Conclusions: Ahmed ClearPath ST250 implantation achieved IOP reduction. These findings suggest that Ahmed ClearPath™ ST250 might be a useful surgical option for the management of refractory glaucoma.

## Introduction

Refractory glaucoma remains a major clinical challenge because inadequate intraocular pressure (IOP) control despite maximal medical and/or surgical treatment may lead to progressive and irreversible visual loss.

Glaucoma drainage devices, including the Ahmed^TM^ Glaucoma Valve (AGV) and Baerveldt^TM^ Glaucoma Implant (BGI), have become established surgical treatment options for refractory glaucoma [1]. Compared with the AGV, the BGI provides superior IOP reduction but has been reported to be associated with a higher incidence of hypotony-related complications [2]. In contrast, the AGV, which incorporates a flow-restrictive valve mechanism, has been associated with a lower incidence of hypotony-related complications but tends to achieve higher postoperative IOP [2]. Accordingly, there has been a need for a novel glaucoma drainage device that combines effective IOP reduction with a lower risk of hypotony-related complications.

To address these limitations, the Ahmed ClearPath^TM^ small tube (ST) was developed as a novel non-valved glaucoma drainage device and is available with plate surface areas of 250 mm² (ST250) and 350 mm² (ST350), similar to the BGI, which is also available in 250-mm² and 350-mm² models. Unlike conventional glaucoma drainage devices such as the AGV and the BGI, both of which have an internal lumen diameter of 305 μm, the Ahmed ClearPath ST incorporates a reduced internal lumen diameter of 127 μm. This smaller lumen is designed to increase early postoperative outflow resistance, thereby reducing the risk of postoperative hypotony while preserving the long-term IOP-lowering efficacy of a non-valved implant. In addition, its low-profile plate and simplified technique may facilitate implantation, and minimize tissue disruption and minimize tissue disruption. The device’s anteriorly positioned suture holes, located 2 mm closer to the limbus than those of the traditional AGV, facilitate easier fixation [3]. However, clinical evidence regarding the safety and efficacy of the Ahmed ClearPath ST250 remains limited.

Therefore, we evaluated the IOP-lowering effect at six months and the safety of Ahmed ClearPath ST250 implantation in patients with refractory glaucoma.

## Materials and methods

This retrospective study was conducted in accordance with the tenets of the Declaration of Helsinki and was approved by the Ethics Committee of Nagoya University Graduate School of Medicine (approval number #2023-023929934). Informed consent was obtained before surgery. Informed consent obtained before surgery included consent for the surgical procedure and for possible participation in future retrospective studies. Consent for participation in this retrospective study was obtained through an opt-out process in accordance with our institutional review board policy. 

Ahmed ClearPath ST250 procedure

One surgeon (KY) performed all surgeries. After routine disinfection, a superior nasal or superior temporal fornix-based conjunctival flap was created, and 2% lidocaine was placed under the Tenon and retrobulbar space. The conjunctiva and Tenon's capsules were carefully dissected to the sclera. After hemostasis, the implant plate was positioned between the rectus muscles and secured to the sclera with nonabsorbable 8-0 nylon sutures 8 mm posterior to the limbus.

The Ahmed ClearPath ST250 (New World Medical, Rancho Cucamonga, USA) is supplied with a preloaded 4-0 polypropylene intraluminal stent, which was retained in all cases to restrict postoperative aqueous outflow. No external tube ligation was performed. A partial-thickness scleral flap was fashioned over the intended tube entry site. The tube was trimmed bevel-up and inserted through a 25-gauge needle tract. For ciliary sulcus insertion, the entry site was created 2.0 mm posterior to the limbus. For pars plana insertion, the entry site was created 3.5 mm posterior to the limbus following a complete pars plana vitrectomy. The tube was secured to the sclera with 10-0 nylon sutures and covered with the scleral flap using 10-0 nylon. Finally, the conjunctiva and Tenon's capsule were closed in a watertight fashion using 8-0 Vicryl sutures. Antimetabolites, such as mitomycin-C, were not used in any case. After surgery, a subconjunctival injection of betamethasone sodium phosphate (2 mg/0.5 mL) was administered, followed by the application of 0.3% ofloxacin ophthalmic ointment and 1% atropine sulfate ophthalmic ointment. 

Postoperatively, topical antibiotics (1.5% levofloxacin hydrate) and steroids (0.1% betamethasone sodium phosphate) were administered four times daily for four weeks.

Information collected for each patient before surgery included age, sex, type of glaucoma, anti-glaucoma medications, type of glaucoma surgery that patients underwent before Ahmed ClearPath surgery, history of intraocular surgery, pre-operative IOP, and pre-operative number of anti-glaucoma medications. All patients were hospitalized postoperatively and observed by the ophthalmologist who performed the surgeries. Postoperative slit-lamp microscopic examination, IOP measurements, best-corrected visual acuity (Log MAR) measurements, and funduscopic examinations were performed on postoperative day 1, at weeks 1 and 2, and at months 1, 3, and 6. IOP was also measured at other time points as clinically indicated. IOP was measured using Goldmann applanation tonometry. In Case 5, IOP was measured using an iCare^TM^ rebound tonometer TA01 (Icare Finland Oy, Vantaa, Finland) because the patient was unable to place her chin on the chin rest. Numerical hypotony was defined as an IOP of <6 mmHg. 

As this was a retrospective study, no prespecified criteria were established; however, the intraluminal stent was generally removed when the IOP exceeded 25 mmHg, regardless of the use of anti-glaucoma medications.

Inclusion and exclusion criteria

Consecutive patients with refractory glaucoma who underwent Ahmed ClearPath ST 250 at Nagoya University Hospital between December 2025 and April 2026 were included. No exclusion criteria were applied.

Refractory glaucoma was defined as glaucoma in eyes with a history of previous glaucoma or vitreoretinal surgery, secondary glaucoma, or glaucoma in patients for whom ophthalmic procedures or follow-up examinations were difficult because of their systemic condition. 

Main outcome

The main outcome measure evaluated in this study was the mean IOP at six months. 

## Results

The demographic characteristics of the patients are shown in Table 1.

**Table 1:** Demographic and clinical characteristics of patients who underwent Ahmed ClearPathTM ST250. M, male; F, female; R, right; L, left; POAG, primary open-angle glaucoma; RRD, rhegmatogenous retinal detachment; gla, glaucoma; DSAEK, Descemet stripping automated endothelial keratoplasty; IOL, intraocular lens; MPCPC, micropulse transscleral cyclophotocoagulation; PFM, PreserFlo^TM^ Microshunt; Pre-ope, pre-operative.

Case	Age (years)	Sex	Eye	Diagnosis	Lens status	Number of previous vitrectomies	Number of previous MPCPC	Number of previous PFM	Number of previous trabeculectomies	Pre-ope IOP (mmHg)	Pre-ope BCVA (Log MAR)
1	47	M	R	Post-vitrectomy for RRD	IOL scleral fixation	4 times + local buckle	1	-	-	30	0.4
2	42	M	R	POAG	IOL	-	-	1	-	40	1.5
3	81	F	L	POAG	IOL	-	-	1	-	32	0.1
4	82	F	L	POAG	IOL	-	-	1	-	40	0.1
5	87	F	R	POAG	IOL	-	1	-	-	45	1.9
6	68	M	R	Uveitic gla	IOL	1	-	-	-	28	1.8
7	74	M	R	Post DSEAK	IOL	1	-	-	3	30	1.4

Seven patients who underwent Ahmed ClearPath^TM ^ST250 were enrolled. Four of the seven patients were male, and three were female. The mean age at the time of surgery was 68.7 ± 17.7 years (average ± standard deviation), and the median age was 74 years (range: 42-87 years). Four patients had primary open-angle glaucoma, one patient had undergone four pars plana vitrectomies and a local buckling procedure for retinal detachment, one had uveitic glaucoma, and one had glaucoma following Descemet stripping automated endothelial keratoplasty. Among the seven eyes, previous glaucoma surgeries included one MPCPC in two eyes, one PreserFlo^TM^ microshunt implantation in three eyes, and three trabeculectomies in one eye. One eye had no history of previous glaucoma surgery. All patients were pseudophakic, and one eye had undergone intrascleral intraocular lens fixation.

Preoperative average IOP was 35.0 ± 6.6 mmHg, and median IOP was 32.0 mmHg (range, 28.0-45.0 mmHg). After Ahmed ClearPathST surgery, IOP decreased to 15.7 ± 9.9 mmHg in one day, median IOP: 12.0 mmHg (range: 5.0-30.0 mmHg), 17.1 ± 12.1 mmHg, median IOP: 11.0 mmHg (range: 6.0-40.0 mmHg) in one week, 13.6 ± 7.5 mmHg, median IOP: 14.0 mmHg (range: 3.0-26.0 mmHg) in two weeks, 10.8 ± 4.1 mmHg, median IOP: 10.5 mmHg (range: 6.0-18.0 mmHg) in one month, 16.2 ± 8.0 mmHg, median IOP: 16.5 mmHg (range: 6.0-28.0 mmHg) in three months, and 11.0 ± 2.7 mmHg, median IOP: 12.0 mmHg (range: 7.0-14.0 mmHg) in six months (Figure 1).

**Figure 1:**
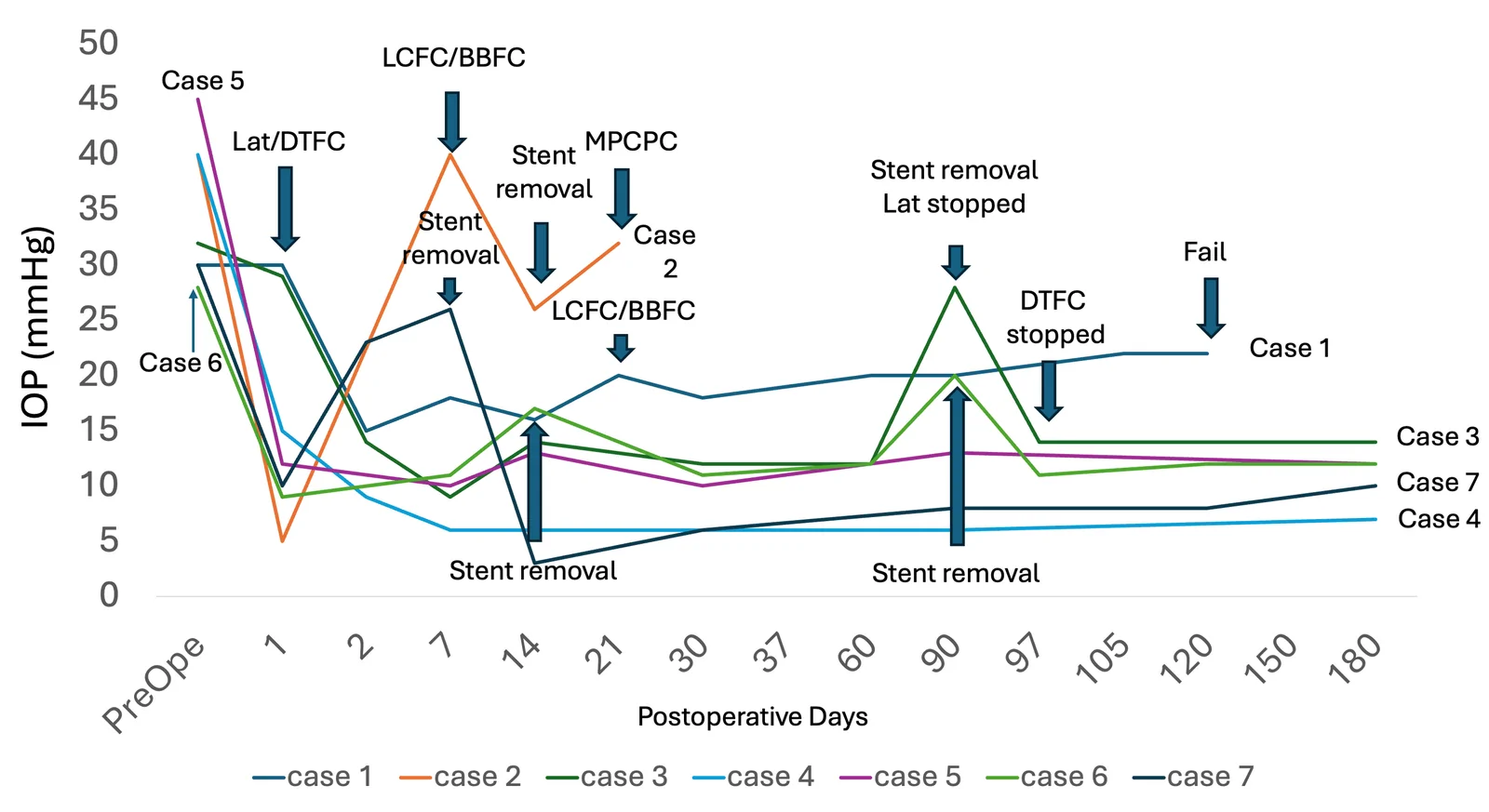
IOP changes, glaucoma medication use, and timing of intraluminal stent removal in individual cases. Lat, latanoprost; DTFC, dorzolamide/timolol fixed combination; LCFC, latanoprost/carteolol fixed combination; BBFC, brinzolamide/brimonidine fixed combination; MPCPC, micropulse transscleral cyclophotocoagulation.

Case 2 underwent MPCPC one month postoperatively and was therefore excluded from subsequent analyses. Case 1 was excluded from the analysis from postoperative month 3 onward because the IOP exceeded 21 mmHg despite medical therapy.

No shallow or flat anterior chamber, choroidal detachment, choroidal haemorrhage, hypotony maculopathy, or conjunctival leak. Numerical hypotony, defined as an IOP of <6 mmHg, was observed in two cases but resolved spontaneously without any specific treatment. No eye movement disorders or diplopia occurred in any of the patients.

Among the seven eyes, stent removal was performed in five eyes on postoperative days 7, 13, 14, 90, and 90, whereas the stent remained in two eyes throughout the follow-up period (Table 2). Cases 4 and 5 did not undergo stent removal because adequate IOP control was maintained with the stent in place. 

**Table 2:** Change in IOP before and after stent removal. Pre-removal IOP was measured immediately before stent removal, and post-removal IOP was measured one week after stent removal. IOP, intraocular pressure

Case	Days from surgery to stent removal	Pre-removal IOP (mmHg)	Post-removal IOP (mmHg)
1	14	20	20
2	13	26	22
3	90	28	14
4	Stent not removed	-	-
5	Stent not removed	-	-
6	90	20	11
7	7	30	3

Clinical course of each case

Case 1

Case 1 was a 47-year-old man with secondary glaucoma in the right eye following multiple vitreoretinal surgeries for rhegmatogenous retinal detachment. He had previously undergone pars plana vitrectomy four times, local scleral buckling, and MPCPC. The eye had an IOL with scleral fixation. The preoperative IOP was 30 mmHg. Ahmed ClearPath ST250 implantation was performed. The Ahmed ClearPath ST250 was implanted in the superonasal quadrant. The tube was inserted beneath the iris into the ciliary sulcus. Gonioscopic examination confirmed that the tube tip was open (Figures 2A, 2B).

**Figure 2:**
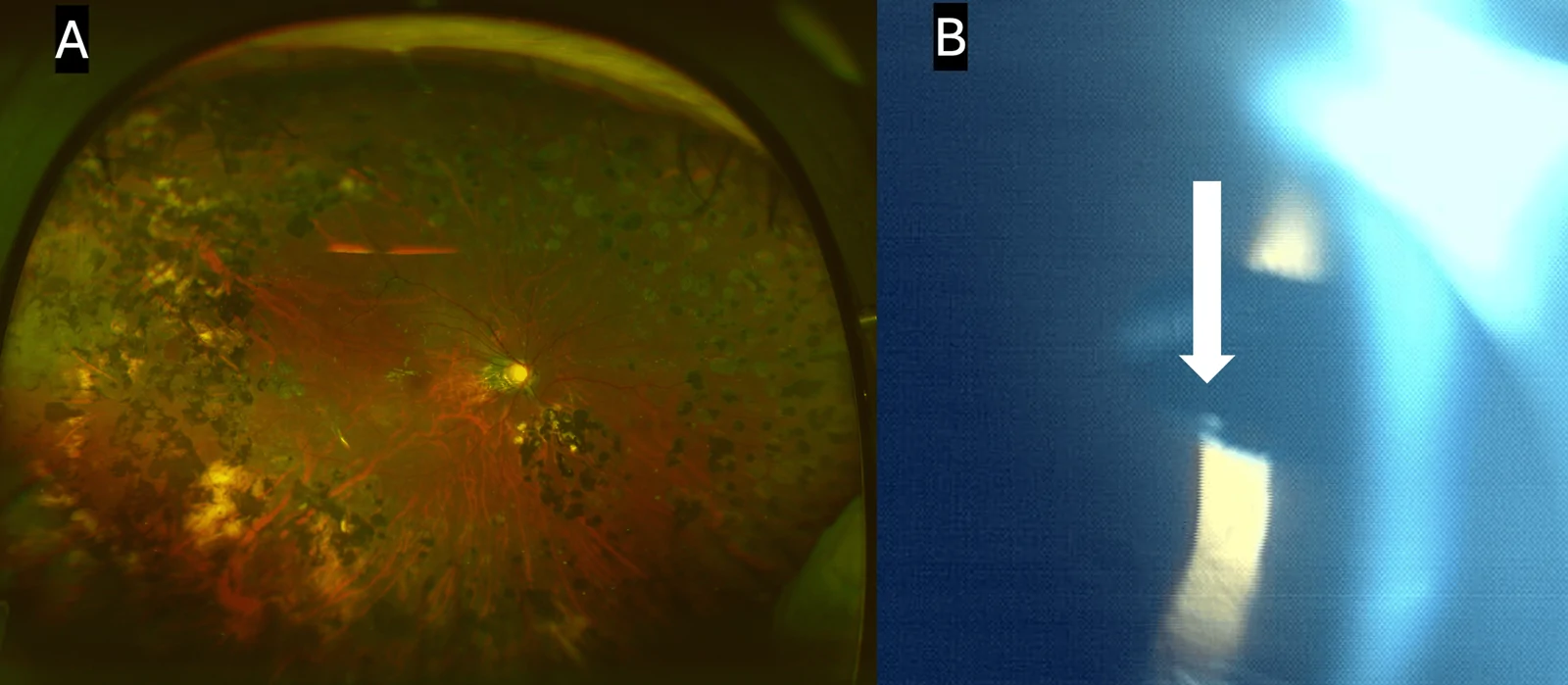
(A) Preoperative fundus photograph. (B) Postoperative gonioscopic image. (A) Preoperative fundus photograph showing retinal photocoagulation scars for a retinal tear and an indentation caused by a local scleral buckle in the inferotemporal quadrant. (B) Postoperative gonioscopic image showing the tip of the tube positioned posterior to the iris.

Postoperative IOP was 30 mmHg on day 1, 18 mmHg at week 1, 16 mmHg at week 2, 18 mmHg at one month, and 20 mmHg at three months. The intraluminal stent was removed 14 days after surgery; the IOP was 20 mmHg immediately before stent removal and 20 mmHg 1 week after removal. Although satisfactory IOP control was not achieved, the IOP remained near the upper limit of the normal range; therefore, no additional intervention was performed. This case was classified as a surgical failure.

Case 2

Case 2 was a 42-year-old man with primary open-angle glaucoma in the right eye. The eye was pseudophakic and had previously undergone PreserFlo MicroShunt surgery. Needling and bleb revision were each performed once for elevated IOP after MicroShunt implantation; however, adequate IOP reduction was not achieved. Preoperative fundus examination revealed marked glaucomatous optic disc cupping. The preoperative IOP was 40 mmHg. The Ahmed ClearPath ST250 was subsequently implanted in the superotemporal quadrant (Figures 3A, 3B).

**Figure 3:**
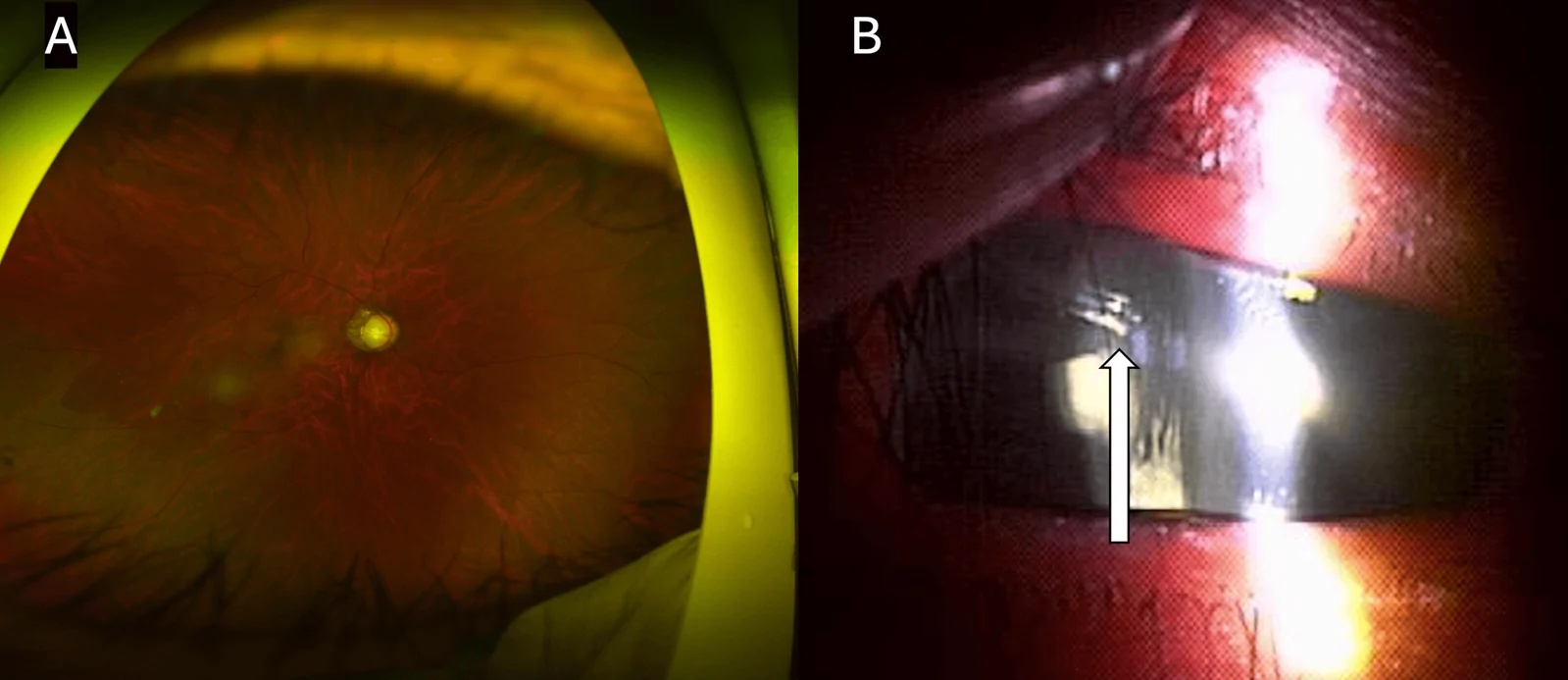
(A) Preoperative fundus photograph. (B) Postoperative slit-lamp photograph. (A) Preoperative fundus photograph showing marked glaucomatous optic disc cupping. (B) Postoperative slit-lamp microscopic image showing the tube inserted into the ciliary sulcus. The white arrow indicates the tube tip.

The tube was inserted into the ciliary sulcus. Postoperative IOP was 5 mmHg on day 1, 40 mmHg at week 1, and 26 mmHg at week 2. The intraluminal stent remained in place throughout this period. This marked fluctuation cannot be fully explained by the flow-restricting mechanism of the stent alone. Although the exact mechanism remains unclear, we speculate that the transient IOP reduction on day 1 may have resulted from peritubular leakage at the tube insertion site, ciliochoroidal detachment, or a transient reduction in aqueous humor production. The intraluminal stent was removed 13 days after surgery; the IOP was 26 mmHg immediately before stent removal and 22 mmHg one week after removal. This case was classified as a surgical failure. MPCPC was performed, resulting in normalization of the IOP.

Case 3

Case 3 was an 81-year-old woman with primary open-angle glaucoma in the left eye. The eye was pseudophakic and had previously undergone PreserFlo MicroShunt surgery. The conjunctiva overlying the MicroShunt had become markedly thinned; therefore, bleb revision was considered difficult. Fundus examination revealed marked glaucomatous optic disc cupping. The preoperative IOP was 32 mmHg. The Ahmed ClearPath ST250 was implanted in the superotemporal quadrant, and the tube was inserted into the ciliary sulcus (Figures 4A, 4B).

**Figure 4:**
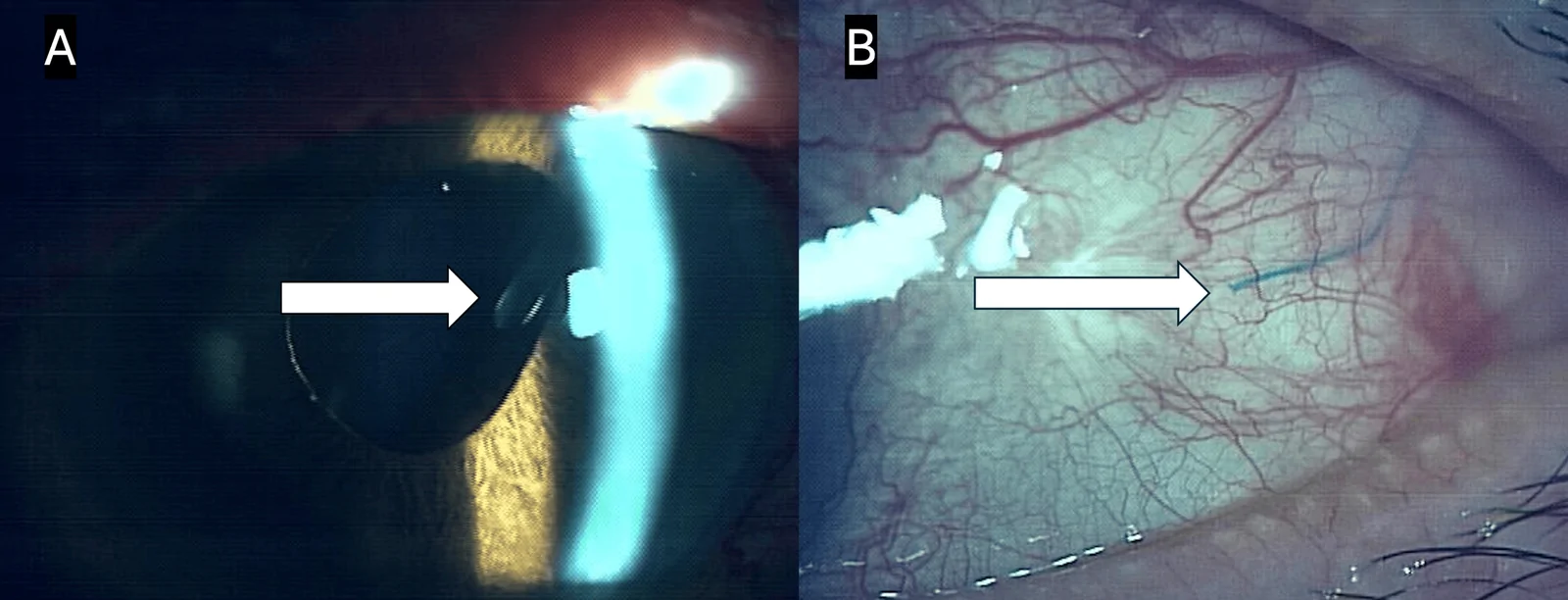
(A and B) Postoperative slit-lamp photographs. (A) Postoperative slit-lamp photograph showing the tube inserted into the ciliary sulcus. The white arrow indicates the tube tip. (B) Slit-lamp photograph obtained immediately before stent removal, showing the stent embedded beneath the conjunctiva. The white arrow indicates the stent.

Postoperative IOP was 29 mmHg on day 1, 9 mmHg at week 1, 14 mmHg at week 2, 12 mmHg at month 1, 28 mmHg at month 3, and 14 mmHg at month 6. The intraluminal stent was removed 90 days after surgery; the IOP was 28 mmHg immediately before stent removal and decreased to 14 mmHg one week after removal. This patient maintained an IOP of approximately 12 mmHg without glaucoma medications.

Case 4

Case 4 was an 82-year-old woman with primary open-angle glaucoma in the left eye. The eye was pseudophakic and had previously undergone PreserFlo MicroShunt surgery. Bleb revision was performed for elevated IOP after MicroShunt implantation; however, adequate IOP control was not achieved. Fundus examination revealed marked glaucomatous optic disc cupping. The preoperative IOP was 40 mmHg. The Ahmed ClearPath ST250 was implanted in the inferotemporal quadrant, and the tube was inserted into the ciliary sulcus. Postoperative IOP was 15 mmHg on day 1, 6 mmHg at week 1, 6 mmHg at week 2, 6 mmHg at month 1, 6 mmHg at month 3, and 7 mmHg at month 6. The intraluminal stent was not removed during the follow-up period. From postoperative week 1 onward, the patient maintained an IOP below 10 mmHg without glaucoma medications (Figures 5A, 5B).

**Figure 5:**
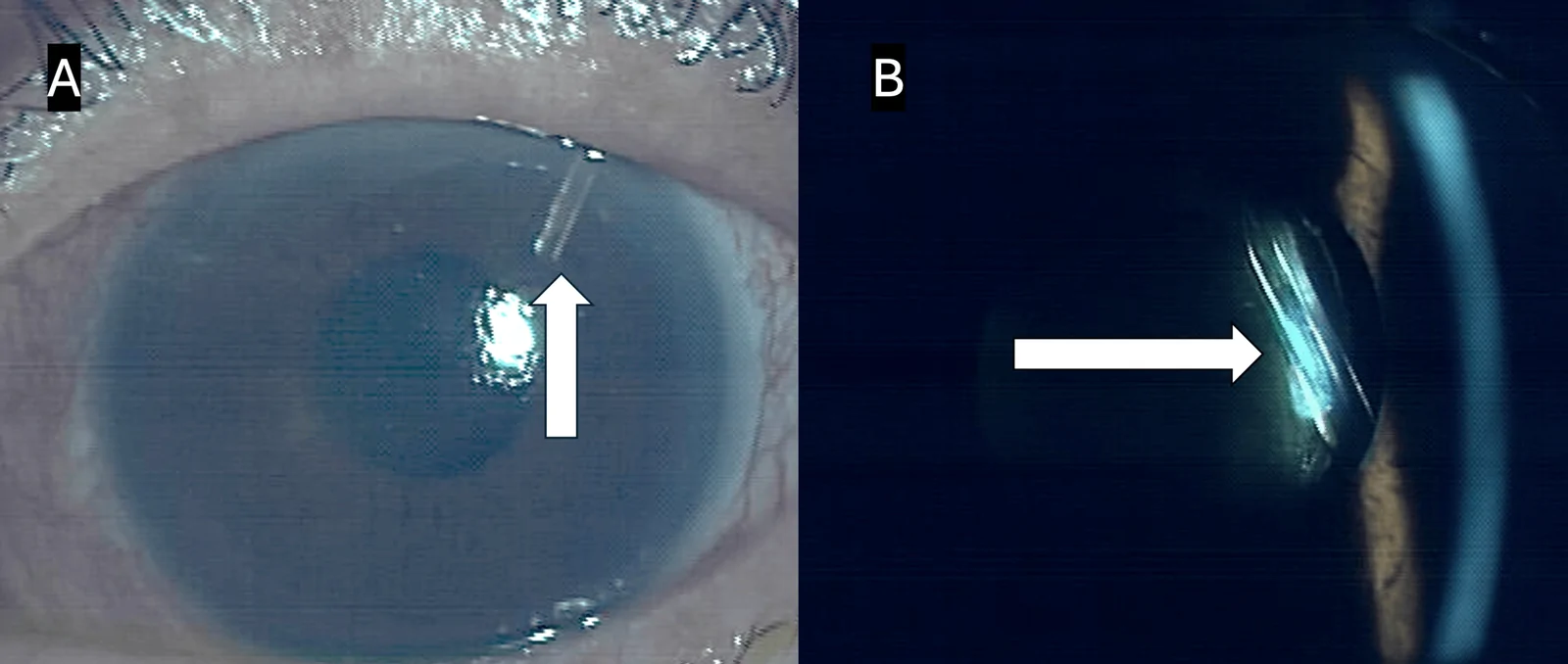
(A) Preoperative slit-lamp photograph. (B) Postoperative slit-lamp photograph. (A) Preoperative slit-lamp photograph showing the previously implanted PreserFlo MicroShunt. The white arrow indicates the PreserFlo MicroShunt. (B) Postoperative slit-lamp photograph showing the tube inserted into the ciliary sulcus in the inferotemporal quadrant. The white arrow indicates the tube tip.

Case 5

Case 5 was an 87-year-old woman with primary open-angle glaucoma in the right eye. The eye was pseudophakic and had previously undergone MPCPC. The preoperative IOP was 45 mmHg. Ahmed ClearPath ST250 implantation was performed (Figures 6A, 6B).

**Figure 6:**
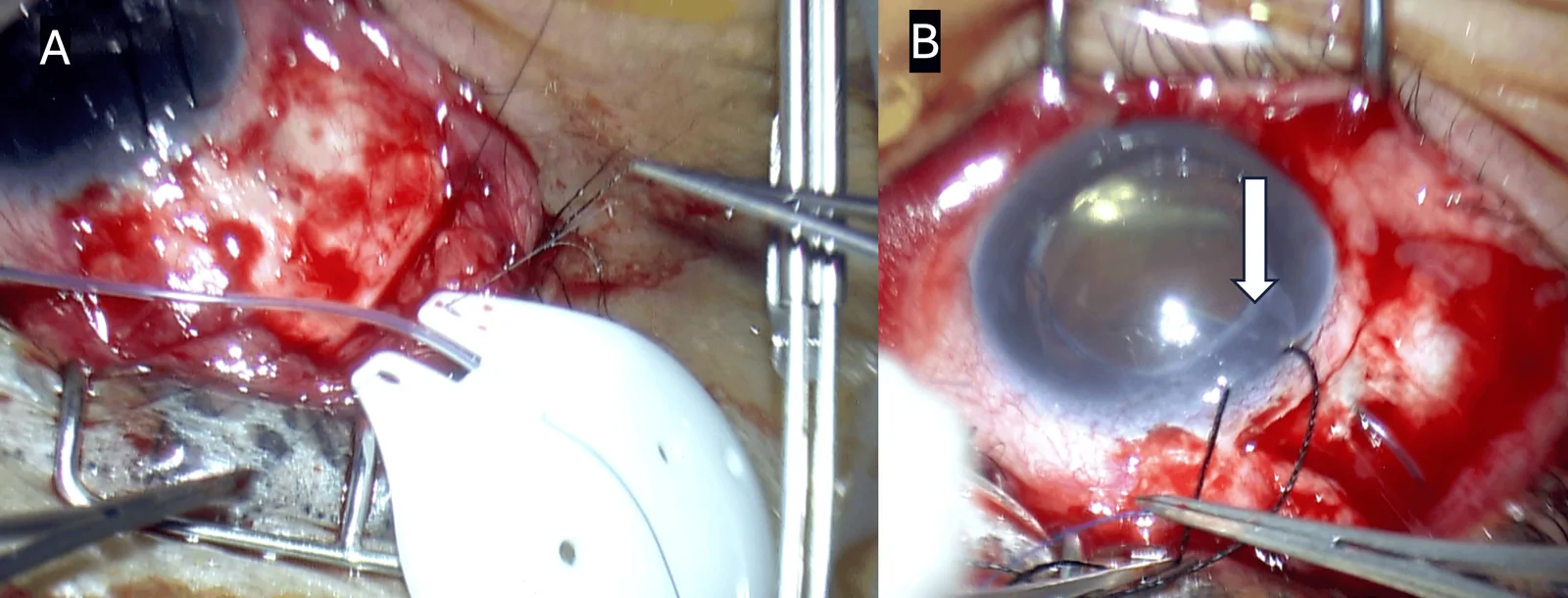
(A) Intraoperative image immediately before implantation of the Ahmed ClearPath™ ST in the superotemporal quadrant. (B) Intraoperative image after implantation of the Ahmed ClearPath™ ST. The tube was inserted into the ciliary sulcus. (A) Intraoperative image immediately before implantation of the Ahmed ClearPath™ ST in the superotemporal quadrant. The ripcord can be observed within the tube. (B) Intraoperative image after implantation of the Ahmed ClearPath™ ST. The tube was inserted into the ciliary sulcus. The white arrow indicates the tube inserted into the ciliary sulcus.

Postoperative IOP was 12 mmHg on day 1, 10 mmHg at week 1, 13 mmHg at week 2, 10 mmHg at month 1, 13 mmHg at month 3, and 12 mmHg at month 6. The intraluminal stent was not removed during the follow-up period. This patient also maintained an IOP of approximately 12 mmHg without glaucoma medications. Case 5 was an elderly wheelchair-dependent woman with a cervical spine disorder. Because she was unable to place her chin on the chin rest of the examination equipment, diagnostic imaging could not be performed. We considered Case 5 to have refractory glaucoma because postoperative follow-up and additional interventions were expected to be difficult, despite the absence of previous glaucoma surgery. In Case 5, IOP was measured using an iCare rebound tonometer. Postoperative fundus examinations confirmed the absence of choroidal detachment and suprachoroidal hemorrhage.

Case 6

Case 6 was a 68-year-old man with uveitic glaucoma in the right eye. The eye was pseudophakic and had previously undergone vitrectomy for epiretinal membrane. The preoperative IOP was 28 mmHg. Ahmed ClearPath ST250 was inserted in the super-temporal area. The tube was inserted into culinary sulcus. Postoperative IOP was 9 mmHg on day 1, 11 mmHg at week 1, 17 mmHg at week 2, 11 mmHg at month 1, 20 mmHg at month 3, and 12 mmHg at month 6. The intraluminal stent was removed 90 days after surgery; the IOP was 20 mmHg immediately before stent removal and decreased to 11 mmHg 1 week after removal. This patient also maintained an IOP of approximately 12 mmHg without glaucoma medications (Figures 7A, 7B). Because the patient had secondary glaucoma associated with uveitis in a previously vitrectomized eye, we considered the glaucoma refractory and selected Ahmed ClearPath™ ST250 implantation as the initial glaucoma surgery. MPCPC was not performed because of the potential risk of reactivating or exacerbating intraocular inflammation. 

**Figure 7:**
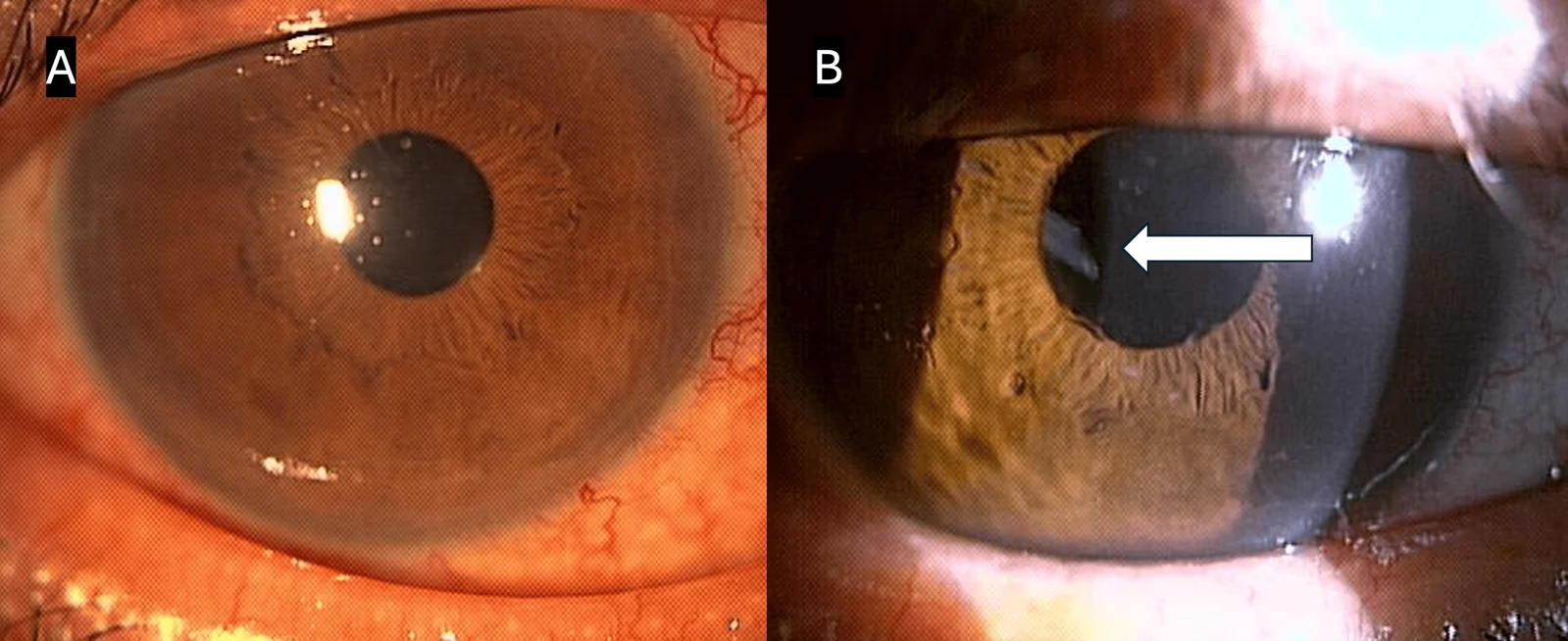
(A) Preoperative slit-lamp photograph. (B) Postoperative slit-lamp photograph. (A) Preoperative slit-lamp photograph showing ciliary injection associated with iritis. (B) Postoperative slit-lamp photograph showing the tube inserted into the ciliary sulcus. The white arrow indicates the tube tip.

Case 7

Case 7 was a 74-year-old man with glaucoma in the right eye following Descemet stripping automated endothelial keratoplasty. The eye was pseudophakic and had previously undergone vitrectomy and three trabeculectomies. In Case 7, the right eye underwent combined trabeculectomy, cataract surgery, and IOL implantation in 2002. A second trabeculectomy was performed in 2006. Vitrectomy was subsequently performed to treat vitreous hemorrhage that developed after this trabeculectomy. A third trabeculectomy was performed later in the same year. DSAEK was performed in 2015, with repeat procedures in 2018 and 2024. Fundus examination showed marked glaucomatous optic disc cupping. The preoperative IOP was 30 mmHg. Ahmed ClearPath ST250 was inserted in the inferotemporal area. The tube was inserted into the vitreous cavity. Postoperative IOP was 10 mmHg on day 1, 26 mmHg at week 1, 3 mmHg at week 2, 8 mmHg at month 1, 10 mmHg at month 3, and 10 mmHg at month 6. The intraluminal stent was removed seven days after surgery; the IOP was 30 mmHg immediately before stent removal and decreased to 3 mmHg 1 week after removal. This patient also maintained an IOP of approximately 12 mmHg without glaucoma medications (Figures 8A, 8B). Numerical hypotony developed after stent removal in this case. The optimal timing of stent removal, as well as gradual, stepwise withdrawal of the stent rather than complete removal at one time, warrants further investigation.

**Figure 8:**
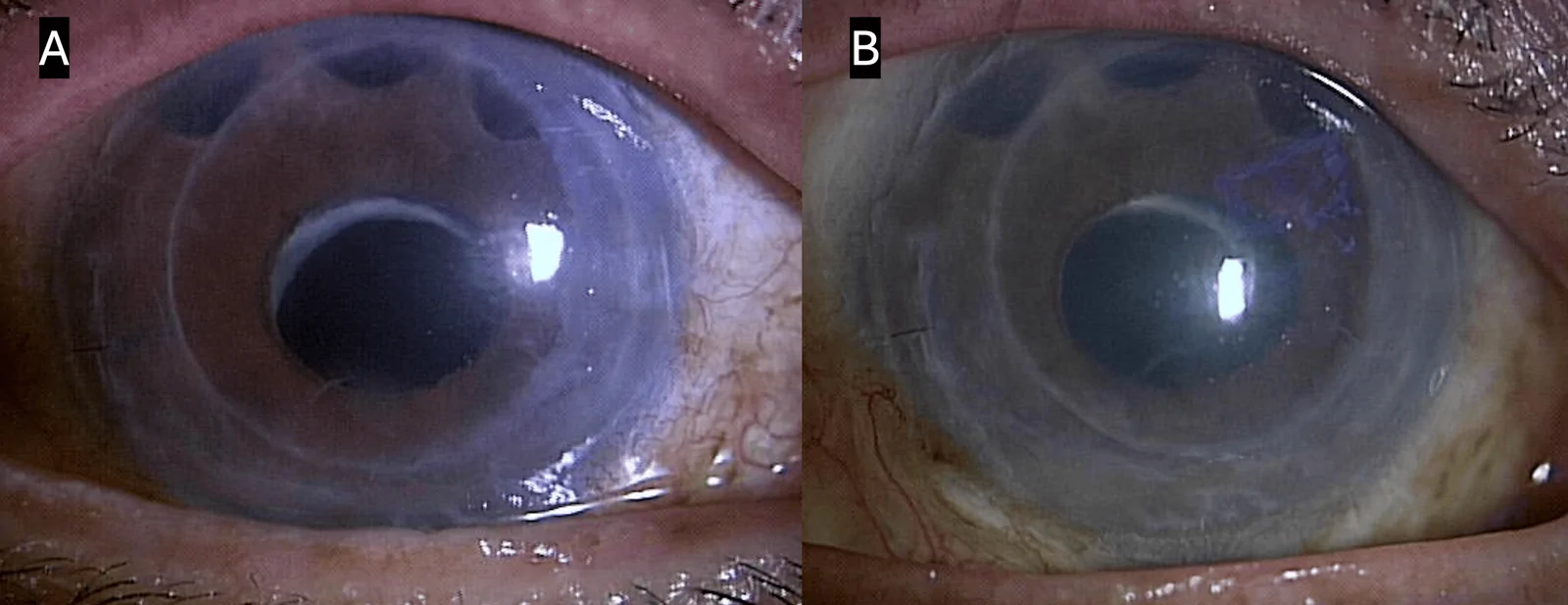
(A) Preoperative slit-lamp photograph. (B) Postoperative slit-lamp photograph. (A) Preoperative slit-lamp photograph showing an eye that had previously undergone Descemet stripping endothelial keratoplasty and three trabeculectomies in the superior quadrant. (B) Postoperative slit-lamp photograph. The tube was inserted into the vitreous cavity.

## Discussion

In this case series, Ahmed ClearPath ST250 implantation reduced the mean IOP from 35.0 ± 6.6 mmHg preoperatively to 11.0 ± 2.7 mmHg at six months while substantially decreasing the number of glaucoma medications. All five patients who achieved successful IOP control were glaucoma medication-free after surgery. No intraoperative or postoperative complications were observed, and surgical success was achieved in 71.4% of eyes. These findings suggest that Ahmed ClearPath ST250 implantation was associated with short-term reductions in IOP and glaucoma medication use in eyes with refractory glaucoma.

In a retrospective study of 17 eyes, Ahmed ClearPath ST implantation significantly reduced mean IOP from 27.2 ± 6.3 mmHg preoperatively to 12.2 ± 5.5 mmHg at six months (55.1% reduction; P < 0.0001). The mean number of glaucoma medications also decreased from 3.35 ± 1.41 to 1.07 ± 0.99 (68.1% reduction; P < 0.0001). Surgical success was achieved in all eyes with six-month follow-up, and no intraoperative complications, postoperative hypotony, or IOP spikes were reported [4]. In this study, the authors did not specify whether the Ahmed ClearPath ST250 or ST350 was implanted. 

The Ahmed ClearPathST is designed with a smaller-lumen tube to reduce the risk of hypotony-related complications. However, clinical evidence regarding the Ahmed ClearPath ST remains limited. Therefore, previous comparative studies evaluating the PAUL Glaucoma Implant (PGI), which also features a small-lumen tube, and the AGV may provide useful insights into the potential clinical performance of the Ahmed ClearPath ST.

Two comparative studies evaluating the PGI and the AGV reported comparable rates of postoperative hypotony despite the absence of a flow-restricting valve in the PGI. In one study of complex glaucoma, numerical hypotony (IOP <6 mmHg) occurred in 2 of 64 PGI eyes (3.1%) and 2 of 40 AGV eyes (5.0%) during an 18-month follow-up, with no significant difference between the groups (P = 1.0) [5]. In another study comparing standalone PGI and AGV implantation, early or late numerical hypotony (IOP <6 mmHg) occurred in 6 of 28 PGI eyes (21.4%) and 4 of 24 AGV eyes (16.6%) respectively, again without a significant difference between the groups (P = 0.87) [6]. 

Although these studies did not evaluate the Ahmed ClearPath ST, the PGI shares the same tube inner diameter (127 μm) as the Ahmed ClearPath ST. Therefore, these findings suggest that reducing the tube lumen alone may provide resistance to aqueous outflow sufficient to achieve a hypotony profile comparable to that of the valved AGV.

In the present study, we used the Ahmed ClearPath ST250. Among currently available small-lumen glaucoma drainage devices, the Ahmed ClearPath ST250 is the only device with a plate surface area of 250 mm². The Ahmed ClearPath ST is also available with a 350-mm² plate, whereas the PGI has a plate surface area of approximately 350 mm². However, no study has directly compared different plate sizes among small-lumen glaucoma drainage devices. Therefore, although the BGI does not have a small-lumen tube, previous studies comparing the BGI-250 and BGI-350 may provide relevant indirect evidence regarding the influence of plate size on surgical outcomes.

In a retrospective comparative study of 89 eyes with refractory glaucoma, including 37 eyes treated with the BGI-250 and 52 eyes treated with the BGI-350, no significant differences were observed between the BGI-250 and BGI-350 in surgical success (P = 0.98), final visual acuity (P = 0.09), IOP (P = 0.23), number of glaucoma medications (P = 0.82), complication rates (P = 0.82), or failure rates (P = 0.64) [7]. Similarly, a retrospective cohort study including 133 eyes treated with BGI-250 and 144 eyes treated with BGI-350 implantations reported cumulative failure rates of 39% and 33%, respectively, at 12 months, with no significant difference between the groups. The mean IOP at 12 months was also comparable between the BGI-250 and BGI-350 groups (13.1 ± 4.6 vs 12.4 ± 4.4 mmHg, P = 0.71) [8]. Kilgore et al. also found no substantial difference in surgical success between the BGI-250 and BGI-350 [9]. Collectively, these studies suggest that increasing the plate surface area from 250 to 350 mm² may not confer a clear advantage in terms of surgical success or IOP reduction. However, in BGI implantation, tube ligation is considered to limit the flow of aqueous humor containing inflammatory cytokines to the area surrounding the plate during the early postoperative period. In contrast, with the Ahmed ClearPath, aqueous humor containing inflammatory cytokines may reach the area surrounding the plate during the early postoperative period. Therefore, postoperative outcomes with the Ahmed ClearPath may not necessarily be comparable to those achieved with the BGI.

A comparative study of Ahmed ClearPath and BGI implantation also included both 250- and 350-mm² models. Approximately 70% of eyes in the Ahmed ClearPath group received the 250-mm² model, whereas approximately 70% of eyes in the BGI group received the 350-mm² model. Although the IOP-lowering effect did not differ significantly between the Ahmed ClearPath and BGI groups, postoperative diplopia occurred in seven eyes, all of which had received a 350-mm² implant, regardless of the implant type [10]. Although this finding does not establish a direct causal relationship between plate size and diplopia, it suggests that a smaller plate may reduce the risk of postoperative diplopia.

Taken together, the available evidence suggests that the Ahmed ClearPath ST250 may provide effective IOP control without an apparent disadvantage associated with its smaller plate and may potentially reduce the risk of postoperative diplopia compared with 350-mm² implants. Nevertheless, evidence regarding the influence of plate size among small-lumen glaucoma drainage devices remains limited, and direct comparative studies of the Ahmed ClearPath ST250, Ahmed ClearPath ST350, and the PGI are warranted.

Although this was a retrospective case series, the stent was generally removed when the IOP exceeded 25 mmHg, irrespective of the use of glaucoma medications. In Cases 1 and 2, the stents were removed on postoperative days 13 and 14, respectively; however, sufficient IOP reduction was not achieved. Both patients were in their 40s, and their relatively young age may have been associated with a more vigorous wound-healing response and earlier fibrotic encapsulation around the plate [11]. This observation raises the possibility that earlier stent removal may be preferable in younger patients, although this hypothesis requires further investigation.

In Case 7, the stent was removed on postoperative day 7 because the IOP had increased to 30 mmHg; however, the IOP subsequently decreased to 3 mmHg. In older patients such as this individual, gradual, stepwise withdrawal of the stent rather than complete removal at one time may help reduce the risk of hypotony. 

Three eyes in this series had previously undergone vitrectomy; however, vitreous cavity insertion was performed in only one eye. Even in previously vitrectomized eyes, residual vitreous may remain and potentially obstruct the tube tip. Therefore, we generally prefer ciliary sulcus insertion, even in eyes with a history of vitrectomy. In addition, obstruction of a tube placed in the ciliary sulcus can be managed using an anterior segment approach, whereas obstruction of a tube placed in the vitreous cavity may require additional vitreous surgery. In Case 7, however, the tube was inserted into the vitreous cavity because the eye had previously undergone corneal transplantation, and minimizing the potential impact on the corneal endothelium was considered particularly important.

Limitations

This study has several limitations. First, its retrospective design is subject to inherent biases, including selection bias. Second, this was a single-center study in which all procedures were performed by a single surgeon, potentially limiting the generalizability of the findings. Third, the sample size was small. Additional limitations of this study include the absence of a control group, heterogeneity in glaucoma diagnoses and previous surgical procedures, variability in the timing of stent removal, and the potential for selection bias inherent in the retrospective study design. In addition, the IOP in Case 4 remained at approximately 6 mmHg during follow-up. The possibility that hypotony-related complications may develop with longer follow-up cannot be excluded. Finally, the follow-up period was limited to short periods, precluding assessment of long-term efficacy and late postoperative complications, such as corneal endothelial cell loss and tube-related complications. Further studies with larger cohorts and longer follow-up are warranted.

## Conclusions

Ahmed ClearPath ST250 implantation resulted in a reduction in IOP, accompanied by a decrease in the number of glaucoma medications, in eyes with refractory glaucoma. Ahmed ClearPath ST250 implantation might provide effective short-term IOP control in refractory glaucoma. However, studies with larger sample sizes and longer follow-up periods are warranted to confirm its long-term efficacy and safety.
